# A Time to Wean? Impact of Weaning Age on Anxiety-Like Behaviour and Stability of Behavioural Traits in Full Adulthood

**DOI:** 10.1371/journal.pone.0167652

**Published:** 2016-12-08

**Authors:** S. Helene Richter, Niklas Kästner, Dirk-Heinz Loddenkemper, Sylvia Kaiser, Norbert Sachser

**Affiliations:** Department of Behavioural Biology, University of Münster, Münster, Germany; UPMC, FRANCE

## Abstract

In mammals, weaning constitutes an important phase in the progression to adulthood. It comprises the termination of suckling and is characterized by several changes in the behaviour of both mother and offspring. Furthermore, numerous studies in rodents have shown that the time point of weaning shapes the behavioural profile of the young. Most of these studies, however, have focused on ‘early weaning’, while relatively little work has been done to study ‘late weaning’ effects. The aim of the present study was therefore to explore behavioural effects of ‘late weaning’, and furthermore to gain insights into modulating effects of weaning age on the consistency of behavioural expressions over time. In total, 25 male and 20 female C57BL/6J mice, weaned after three (W3) or four (W4) weeks of age, were subjected to a series of behavioural paradigms widely used to assess anxiety-like behaviour, exploratory locomotion, and nest building performance. Behavioural testing took place with the mice reaching an age of 20 weeks and was repeated eight weeks later to investigate the stability of behavioural expressions over time. At the group level, W4 mice behaved less anxious and more explorative than W3 animals in the Open Field and Novel Cage, while anxiety-like behaviour on the Elevated Plus Maze was modulated by a weaning-age-by-sex interaction. Furthermore, weaning age shaped the degree of behavioural stability over time in a sex-specific way. While W3 females and W4 males displayed a remarkable degree of behavioural stability over time, no such patterns were observed in W3 males and W4 females. Adding to the existing literature, we could thus confirm that effects of weaning age do indeed exist when prolonging this phase, and were furthermore able to provide first evidence for the impact of weaning age and sex on the consistency of behavioural expressions over time.

## Introduction

In mammals, weaning constitutes an essential phase in the progression to adulthood. It describes the infant's transition from complete dependence of the mother to nutritional and social independence and is accompanied by marked changes in the behaviour and the physiology of both mother and offspring. While it has been suggested that the complete termination of suckling is a key characteristic of this phase, it seems far more difficult if not impossible to determine the exact beginning and end of the weaning process in most species [1, 2]. Weaning is thus not simply an event, but a gradual process that is furthermore subject to numerous environmental or social influences. From an ecological perspective, the process of weaning has therefore been identified as a main life-history variable that is crucially involved in both sexual and reproductive strategies [3, 4].

Much of the early work on weaning has been done in house mice that are characterized by their fast reproduction and remarkable adaptive ability [5]. As it is typical for an altricial species, new-born mice are hairless, blind, deaf, and have undeveloped motor skills, making them fully dependent on their mother for nutrition and thermoregulation [6]. As they grow older, pups become increasingly independent and start to shift to solid food. Simultaneously, a sharp decrease in maternal investment can be observed that indicates the beginning of the weaning phase. Under natural conditions, the process of weaning commences around day 17 of lactation and is completed after 20 to 23 days [7–9]. However, dependent on the litter size and the physical development of the young, weaning can also be prolonged until days 30 to 35 postpartum [10]. By contrast, laboratory weaning in mice is often carried out on a single day, by separating the mother abruptly from its litter. Such artificial weaning routines include mother-offspring separation at various time points between postnatal days 18 and 28, but mostly take place around postnatal day 21. This is mainly due to the fact that so-called ‘continuous mating strategies’ are adopted to maximize the breeding success and the productivity in big breeding units [11]. Here, mating takes place during postpartum oestrus, so that the following gestation period of about 21 days completely overlaps with raising the young of the previous litter. Weaning under laboratory conditions is thus mainly determined by practical rather than ecological considerations.

However, there is converging evidence from numerous studies on rodents that experiences made during early phases of life can have profound effects on the later behavioural profile (for review see [12–14]). For example, extensive research on rats has shown that daily separation of the mother from the pups for either prolonged or brief periods during the first one or two weeks are associated with persistent changes in the physiology and the behaviour of the offspring (for review see [15]). Similarly, natural variations in maternal care, such as individual differences in the level of licking/grooming, have been found to induce long-term changes in gene expression or behavioural profiles of the young (e.g., [16–18]). Given that early interactions among parents and siblings thus seem to play a key role in socially mediated behavioural plasticity, the weaning process may represent another sensitive phase in development. Indeed, several studies in mice and rats could show that the time point of weaning influences the physiological and neurobehavioural development of the young. For example, ‘early weaned’ mice that were separated from the mother at the age of 14 or 15 days, displayed more anxiety-like behaviour [19, 20], increased locomotor activity [21], and higher levels of aggression [22] in comparison to mice weaned at the age of 21 days. With respect to physiological and neural correlates, ‘early weaning’ was associated with an elevated neuroendocrine response to mild stress [23], decreased BDNF concentrations in the hippocampus [24], and altered myelin formation in the brain during the developmental period [25]. Notably, most of these weaning studies focused on the impact of ‘early weaning’, while far less is known about effects of ‘late weaning’ on the offspring development. However, in contrast to an ‘early weaning’ treatment that partly overlaps with aspects of ‘maternal deprivation’, ‘late weaning’ may better represent the natural and hence gradual process of weaning. Some first results in mice indicate that such a ‘late weaning’ after four weeks of age also exerts effects on both behavioural and neural correlates. In particular, extending the duration of contact between mother and pups up to 28 days postpartum was found to induce changes in exploratory, social, and maternal behaviour, and was, furthermore, associated with altered levels of oxytocin and vasopressin V1a receptor density in various brain areas in a sex-specific way [26].

During the past decade, the study of animal behaviour has undergone a major shift. In addition to simply comparing population or sample means, inter-individual differences that traditionally were considered as noise have become a key target of research approaches [27, 28]. Referred to as ‘animal personalities’ in the literature, such inter-individual differences that are consistent over time and/or across contexts have meanwhile been investigated in numerous taxa, including fish, birds, reptiles, and mammals (for review see [29]). While it is thus now widely accepted that stable personality traits do exist in various species, far less is known about factors influencing the stability of such traits. In particular, the questions arise whether such personalities are stable from birth to death or whether the stability changes as a result of past or present experiences [30].

Against this background, the aims of the present study were twofold: First, by comparing mice weaned after either three or four weeks of age, we aimed at investigating the consequences of ‘late weaning’ on the behavioural profile of male and female C57BL/6J mice. Second, by taking individual differences into account, we intended to provide the first examination of the influence of weaning age on behavioural stability over time in full adulthood. By conducting a series of four standard behavioural tests twice at an interval of eight weeks, we expected to find differences in anxiety-like behaviour, exploratory locomotion, and nest building performance between male and female mice with different weaning experiences. Furthermore, we hypothesized to find differences in the behavioural stability over time between mice that were weaned after three or four weeks of age, respectively.

## Animals and methods

### Animals and housing conditions

Twenty-four female mice of the inbred strain C57BL/6J were purchased from a professional breeder (Charles River Laboratories, Research Models and Services, Germany GmbH, Sulzfeld, Germany) in the second week of pregnancy. Out of these animals, 21 females gave birth and successfully reared pups. Litter sizes ranged from 4 to 9 pups. These litters were assigned to two weaning treatments with the pups being weaned after either three (W3 litters, N = 11) or four weeks of age (W4 litters, N = 10). Allocation to treatment groups was balanced with respect to litter size to avoid any systematic differences between litters selected for the W3 treatment and those for the W4 treatment. For the subsequent behavioural investigation, 25 male (N_W3_ = 12, N_W4_ = 13) and 20 female pups (N_W3_ = 10, N_W4_ = 10) were randomly selected from these litters.

Upon arrival, dams were housed individually in standard Makrolon cages type III (37 x 21 x 15 cm^3^) with wood shavings as bedding material (Allspan, Höveler GmbH & Co. KG, Langenfeld, Germany), a semi-transparent red plastic house (11.1 x 11.1 x 5.5 cm^3^, Tecniplast Deutschland GmbH, Hohenpeißenberg, Germany), a wooden climbing frame, and a cotton nestlet (ZOONLAB GmbH, Castrop-Rauxel, Germany) as enrichment. Mouse diet (Altromin 1314; Altromin Spezialfutter GmbH & Co. KG, Lage, Germany) as well as water were available *ad libitum*. Cages were cleaned on a weekly basis, however not within the first five days after the birth of the young. The enrichment was exchanged on a fortnightly basis. The colony room was maintained at a temperature of about 23°C, a relative humidity of about 50% and a reversed 12 h light-dark cycle (lights off at 10 a.m.). At weaning, pups were weighed and allocated to same-sex sibling groups of two to five individuals per cage. Housing groups thereby consisted of one experimental subject and one to four same-sex siblings that were not included in the experiment. Housing conditions were the same as for the dams, however, different diet was provided (Altromin 1324; Altromin Spezialfutter GmbH & Co. KG, Lage, Germany) and the wooden climbing frame was exchanged by a wooden stick.

### Ethics statement

All procedures complied with the regulations covering animal experimentation within the EU (European Communities Council DIRECTIVE 2010/63/EU) and were approved by the national and local authorities (Landesamt für Natur, Umwelt und Verbraucherschutz Nordrhein-Westfalen “LANUV NRW”, reference number: 84–02.05.20.12.212). Our study involved behavioural testing only and did not cause any distress or pain to the animals. After the study, the animals remained in the animal facility of the institute for further behavioural studies.

### Experimental design

**Fig 1:**
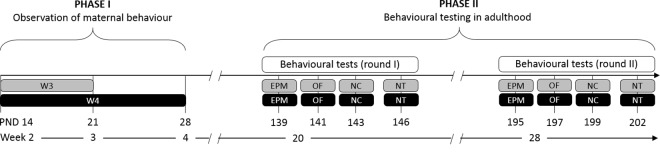
Experimental design. The experiment was split into two phases: 1) Observation of maternal behaviour during weeks 3 and 4, and 2) behavioural testing of the offspring in full adulthood. Male and female C57BL/6J mice were weaned after either three (W3) or four (W4) weeks of age and housed in same-sex groups of two to five siblings per cage. To account for litter effects in the experimental design, only one or two subjects per sex and litter were included in the experiment. At the age of 20 weeks, mice were subjected to the first round of behavioural tests, including Elevated Plus Maze (EPM), Open Field (OF), Novel Cage (NC), and Nest Test (NT). To investigate behavioural stability over time, the same tests were repeated at an interval of eight weeks with the animals reaching an age of about 28 weeks.

The experiment was conducted in two independent replicates over a period of about 9 months. Per replicate the experimental procedure was split into two phases: 1) Observation of maternal behaviour and 2) behavioural testing of W3 and W4 offspring in full adulthood (Fig 1). Maternal behaviour was observed from postnatal day 15 to 21 in all dams (week 3, N = 21) and from postnatal day 22 to 28 in dams of the W4-litters (week 4, N = 10). After weaning, mice were left undisturbed until the start of behavioural testing. To investigate effects of the weaning age on the behavioural profile in full adulthood, male and female offspring underwent a battery of standard behavioural tests between PNDs 139 ± 2 and 147 ± 2 (test round I): Elevated Plus Maze (PND 139 ± 2), Open Field (PND 141 ± 2), and Novel Cage (PND 143 ± 2) as common paradigms to assess anxiety-like behaviour and exploratory locomotion as well as the Nest Test (PND 146 ± 2) to study the individuals’ nest-building performance (Fig 1). To measure behavioural stability over time, these tests were then repeated after 8 weeks (test round II), i.e. between PNDs 195 ± 2 and 202 ± 2. In addition to the behavioural investigation, body weights were assessed at 6, 12, 18, 24 and 30 weeks of age to detect effects of weaning age on the later physical development.

#### Maternal behaviour

Methods for the investigation of maternal behaviour were adopted from previous studies on rats and mice [26, 31–33]. Briefly, observations took place under red light conditions during the dark period, i.e. during the active phase of the animals. In total, four observation sessions of one hour each were conducted per day, starting at 10:30 am, 12:45 am, 3:00 pm, and 5:15 pm. During each observation session, a cage was scanned every 3 minutes, leading to a total amount of 20 scans per hour and 80 scans per dam and day. Altogether, seven behaviours were recorded by applying scan sampling and instantaneous recording [34] that were defined according to established protocols ([26, 33, 35], for ethogram see Table 1). Owing to observational problems in the enriched cages, absolute observed frequencies were corrected for the total amount of scans a mouse was visible during the observation sessions for the analysis.

**Table 1:** Definitions of maternal behaviours.

Behaviour	Definition
Nursing	Lying motionless or in a crouched arch posture over the pups, at least one pup is attached to the dam’s nipples
Contact with pups	Being in physical contact to at least one pup without grooming, licking or nursing for longer than 1 sec
Eating	Chewing food pellets
Drinking	Mouth being in contact with spout of the water bottle
Nest building	Moving bedding around the pups with the snout and/or the paws
Self-grooming	Licking, scratching or brushing own fur with tongue or paws
Licking/grooming	Touching any part of a pup's body with the tongue or nose or forepaws, but no nursing occurring

#### Behavioural testing

Adult mice were tested successively in three behavioural paradigms for anxiety-like behaviour and exploratory locomotion (i.e. Elevated Plus Maze, Open Field, Novel Cage). Furthermore, as nests are highly important for temperature regulation as well as for reproduction and shelter, nest building performance was investigated in the Nest Test [36]. Between the individual tests, there was an interval of at least 48 h. The tests on anxiety-like behaviour and exploratory locomotion were performed during the dark period, i.e. the animal’s active phase, between 10:30 am and 4:00 pm, while the Nest Test was started at 9:00 am, i.e. one hour before the beginning of the dark phase, to score the nests at 4:00 pm. Male and female mice were tested consecutively according to a randomized daily order. Thereby, males were always tested before females. A possible experimenter bias was prevented by running the whole experiment as a blind study. Elevated Plus Maze and Open Field were performed in a room different from the subject’s housing room, where subjects were transported individually in an empty polycarbonate type II cage. Behaviour was recorded by a camera (Logitech Webcam Pro 9000) and the animal’s movements were automatically tracked by the software ANY-maze (version 4.75, Stoelting Co., Wood Dale, USA). Novel Cage and the Nest Test scoring took place in the subject’s housing room under red light conditions and behaviour was recorded via direct observations. All equipment used during the procedures was cleaned with 70% ethanol and dried between subjects.

**Elevated Plus Maze (EPM):** The maze in the shape of a plus comprised four arms (30 cm x 5 cm each) as well as a central square (5 cm x 5 cm) and was elevated 50 cm above the floor. While two opposite arms were surrounded by 20 cm high walls (closed arms), the two remaining arms were enclosed by a wall of only 0.4 cm height in order to prevent mice from falling off the maze (open arms). The apparatus was constructed of wood painted grey and the surface of the maze was covered by a grey inlay made of PVC. The illumination level in the centre square was set to 25 lux.

To ensure a similar level of activation, mice stayed in their transport cages for 1 minute and were then placed in the centre square of the EPM with the head in direction of always the same closed arm. The experimenter left the room and the apparatus could be explored freely by the mouse for 5 min. Measures taken to assess anxiety-like behaviour were relative time on open arms (time on open arms/ (time on open + time on closed arms)), relative number of open arm entries (open arm entries/ (open arm entries + closed arm entries)) and the distance travelled on the open arms. To assess exploratory locomotion the total path travelled on the maze was recorded (e.g., [37, 38]).

**Open Field (OF):** The apparatus made of white coated plywood consisted of a square arena (80 cm x 80 cm) surrounded by walls (42 cm). Illumination level in the centre was set to 25 lux. Subjects were placed in a black cylinder (diameter: 11 cm, height: 28 cm) located in always the same corner of the arena. The cylinder was lifted after 1 min, the experimenter left the room, and the apparatus could be explored freely by the mouse for 5 min. Measures taken to assess anxiety-like behaviour were time in the centre (defined as the area being located at least 20 cm distant from the walls), number of centre entries, and the distance travelled in the centre. Exploratory locomotion was assessed on the basis of the total distance travelled within the complete arena (e.g., [37, 38]).

**Novel Cage (NC):** Subjects were placed into always the same corner of a standard Makrolon cage type III with a thin layer of bedding material and allowed to explore the cage freely for 5 min. As a measure for exploratory locomotion, the number of rearing, defined as the mouse raising itself on its hind paws and stretching its snout into the air, was recorded (e.g., [39–41]).

**Nest Test (NT):** Subjects were introduced into a new Makrolon type III cage, equipped with a thin layer of bedding material, water and pellet diet *ad libitum*. Additionally, one cotton nestlet (5 cm x 5 cm, ZOONLAB GmbH, Castrop-Rauxel, Germany) was provided as nesting material. Cages were placed in racks and left undisturbed for the complete testing phase. Nest building performance was scored after 7 h, using a scale adopted from Deacon [36], ranging from 0 (nestlet completely intact) to 5 (> 90% of the nestlet shredded, near perfect nest with walls higher than the mouse).

### Data analysis

Since the behavioural investigation followed in the wake of an early postnatal, and as such litter-dependent maternal, treatment, the statistical analysis was sensitive to litter effects [42]. To account for these, one or two male or female pups from the same litter were included in the experiment as the assessment of additional pups beyond the first two pups may not provide additional statistical power [43, 44].

Maternal behaviour as well as the offspring’s performance in the behavioural tests and the body weights were analysed using parametric statistics. Therefore, residuals were examined graphically for homoscedasticity and outliers, and the Kolmogorov-Smirnov and Shapiro-Wilk tests were applied to check for normal distribution. When necessary, the raw data were transformed using square-root or logarithmic transformations (square-root: Elevated Plus Maze, open arm distance; log: Open Field, centre time).

In particular, maternal behaviour was compared between W3 and W4 dams using univariate ANCOVAs with ‘weaning age’ as fixed factor and ‘litter size’ as covariate. Additionally, litter sizes were compared between the groups using a t-test for independent samples. To investigate effects of the time point of weaning on the offspring’s behaviour, behavioural parameters were analysed conducting two-way ANOVAs with ‘weaning age’ (W3, W4) and ‘sex’ (male, female) as fixed factors. For the analysis of body weights a repeated measures ANCOVA was performed with ‘time’ (weeks 6, 12, 18, 24, 30) as within-subjects factor and ‘weaning age’ and ‘sex’ as between-subjects factors. As larger litters have on average smaller offspring, ‘litter size’ was additionally included as ‘covariate’ in all body weight analyses. In order to account for possible violations to sphericity, the Greenhouse-Geisser correction was applied.

Behavioural stability over time was investigated in untransformed data by calculating Spearman’s rank correlation coefficient (1-tailed). Thus, multiple correlations were run to analyse the relationship between the behavioural performance in test round I and test round II, assuming that an animal with a high performance in test round one is also characterized by a high performance in test round two (= behavioural stability). All statistical analyses were carried out using SPSS 22 (SPSS Inc., Chicago, IL, U.S.A.), and differences were considered to be significant at p < 0.05. P-values between 0.05 and 0.1 were set as statistical trends.

## Results

### Litter size and maternal behaviour

All litters consisted of at least four pups with an average litter size of 6.67 ± 0.35 (W3: 6.27 ± 0.52; W4: 7.10 ± 0.43). Comparison of litter sizes at birth did not reveal any significant difference between litters selected for weaning after either three (W3) or four (W4) weeks (t-test for independent samples, t_(19)_ = -1.203, p = 0.244).

**Fig 2:**
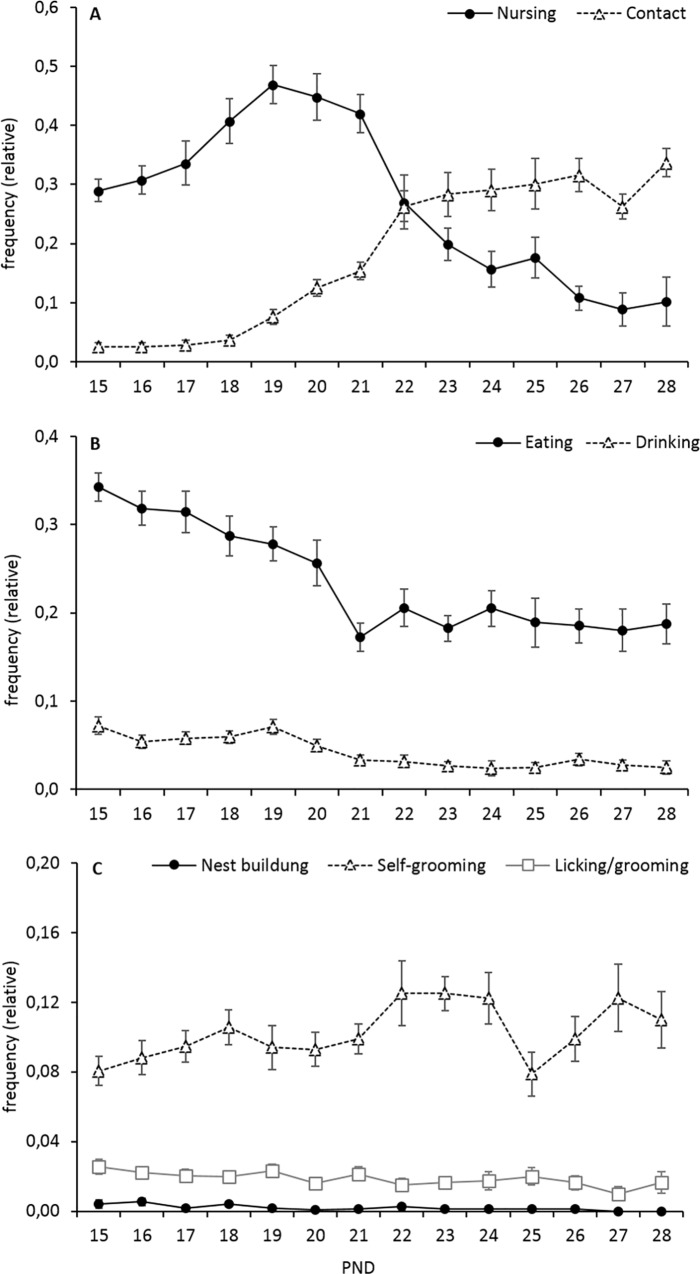
Maternal behaviour in C57BL/6J dams from postnatal days (PND) 15 to 28. (**A**) The frequency of nursing decreased over time, while the frequency of non-nursing contact increased steadily. (**B**) The frequencies of eating and drinking displayed by the dam decreased as pups started to switch to eating solid food. (**C**) Frequencies of nest building and licking/grooming remained constant throughout this period, while the frequency of self-grooming appeared to be slightly increased in the second half of the observation period. Data are presented as means ± SEM. Samples sizes PNDs 15–21 (W3 and W4 dams): n = 21, PNDs 22–28 (W4 dams only): n = 10.

Maternal behaviour was observed from postnatal day 15 to 21 in all dams (week 3, n = 21) and from postnatal day 22 to 28 in dams of the W4 litters (week 4, n = 10). As summarized in Fig 2, the frequency of nursing decreased across time, while the frequency of non-nursing contact increased steadily (Fig 2A). Interestingly, nursing could be observed throughout week 4 with a share of about 10% at the end of the observation period. The frequencies of eating and drinking displayed by the dam were found to decrease as the pups started to switch to eating solid food (Fig 2B). Furthermore, the frequencies of nest building and licking/grooming did not change over time, while the frequency of self-grooming appeared to be slightly higher in week 4 than in week 3 (Fig 2C).

Furthermore, to exclude any systematic differences in maternal care between W3 and W4 dams, maternal behaviour in the third week postpartum was averaged per dam and compared between W3 and W4 dams. As expected, mean frequencies of maternal behaviour did not differ significantly between the groups in week 3 despite of two statistical trends in different directions with respect to nursing and contact with pups (F_(1,18)_ = 0.101–3.751, p = 0.069–0.571, Table 2).

**Table 2:** Maternal behaviour.

	W3 dams			W4 dams			Statistics	
	Mean	±	SEM	Mean	±	SEM	F-ratio	p-value
Nursing (relative)	0.402	±	0.023	0.361	±	0.026	3.527	*0*.*077*
Contact with pups (relative)	0.059	±	0.006	0.077	±	0.008	3.751	*0*.*069*
Eating (relative)	0.269	±	0.018	0.295	±	0.022	0.333	0.571
Drinking (relative	0.058	±	0.008	0.055	±	0.007	1.950	0.180
Nest building (relative)	0.002	±	0.001	0.004	±	0.002	2.307	0.146
Self-grooming (relative)	0.093	±	0.009	0.095	±	0.010	1.017	0.327
Grooming/licking (relative)	0.023	±	0.003	0.020	±	0.002	0.101	0.754

Average maternal behaviour displayed in the third week postpartum by dams raising pups for either three (W3 dams) or four (W4 dams) weeks. Data are presented as means ± SEM and statistical findings of the ANCOVAs are summarized by means of p-values and F-ratios. Statistical trends are indicated by italic numbers. Sample sizes: N_W3-dams_ = 11, N_W4-dams_ = 10.

### Body weight development in W3 and W4 offspring

**Fig 3:**
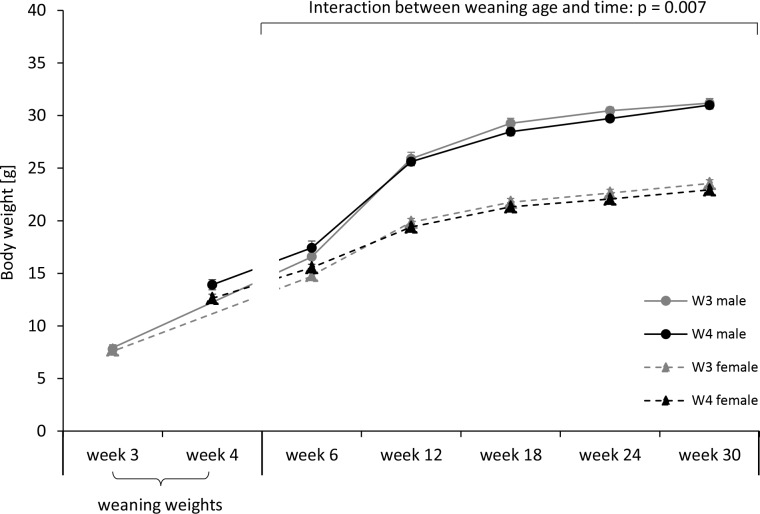
Body weight development in male and female C57BL/6J mice weaned after either three (W3) or four weeks of age (W4). Besides a significant effect of sex and a sex-by-time interaction, a significant interaction between weaning age and time indicated a differential body weight development in W3 and W4 offspring over the course of the experiment. Data are presented as means ± SEM. Statistics: Repeated measures ANCOVA (including only data from week 6 to week 30). Sample sizes: N_males_W3_ = 12, N_females_W3_ = 10, N_males_W4_ = 13, N_females_W4_ = 10.

Not surprisingly, weaning weights differed significantly between mice of the W3 and the W4 groups (2-way ANCOVA, F_(1,40)_ = 174.995, p < 0.001). With an average weaning weight of 13.272 ± 0.291 grams W4 mice were significantly heavier than the younger W3 pups that had an average weaning weight of 7.760 ± 0.296 grams. Males and females did not differ in their weaning weights (F_(1,40)_ = 2.662, p = 0.111). As they grew older, mice steadily gained weight (repeated measures ANCOVA, F_(2.045,81.804)_ = 33.317, p < 0.001) with an increasing weight difference between male and female mice (Fig 3). Statistically, this was confirmed by a significant interaction effect between sex and time (F_(2.045,81.804)_ = 84.497, p < 0.001) as well as by a significant main effect of sex (F_(1,40)_ = 276.696, p < 0.001). Interestingly, a significant interaction between weaning age and time also indicated a differential body weight development in W3 and W4 offspring over the course of the experiment (F_(2.045,81.804)_ = 5.206, p = 0.007). A further graphical examination of this effect revealed that W3 mice were slightly lighter as their W4 conspecifics at the age of six weeks, but heavier from week 12 onwards (Fig 3).

### Anxiety-like behaviour, exploratory locomotion, and nest building performance in W3 and W4 offspring

**Fig 4:**
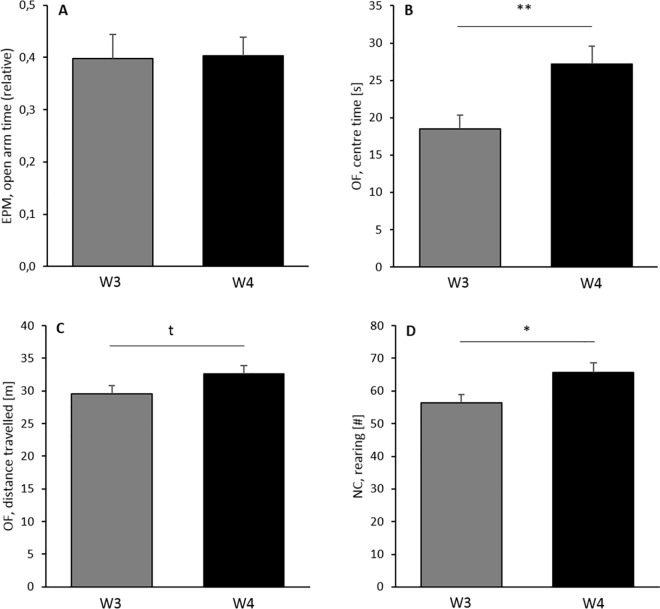
Performance of C57BL/6J mice weaned after either three weeks (W3) or four weeks (W4) of age in a battery of behavioural tests conducted at the age of about 20 weeks. (**A**) Relative time on open arms of the Elevated Plus Maze (EPM), (**B**) time spent in the centre of the Open Field (OF), (**C**) total path travelled in the OF, and (**D**) number of rearings displayed in the Novel Cage (NC). Data are presented as means + SEM. Statistics: 2-way ANOVA, ^t^P < 0.1, *P < 0.05, **P < 0.01, sample sizes: N_W3_ = 22, N_W4_ = 23.

All mice were tested for anxiety-like behaviour, exploratory locomotion, and nest building performance at the age of about 20 weeks, which can be considered as full adulthood. Concerning anxiety-like behaviour, weaning age significantly affected anxiety-like behaviour in the Open Field (OF), but not on the Elevated Plus Maze (EPM, Table 3, Fig 4). In particular, W4 mice entered the centre of the OF significantly more often (2-way ANOVA, F_(1,41)_ = 7.391, p = 0.010), spent more time in the centre (F_(1,41)_ = 8.974, p = 0.005, Fig 4B), and travelled greater distances there than their W3 conspecifics (F_(1,41)_ = 9.459, p = 0.004). On the EPM, however, no differences between W3 and W4 animals were found with respect to relative open arm entries, relative time on the open arms (Fig 4A), and the distance travelled on the open arms (F_(1,41)_ = 0.003–0.459, p = 0.604–0.943, Table 3). Similarly, differences in exploratory locomotion were found in the OF and the Novel Cage (NC), but not on the EPM. Thus, W4 mice tended to travel greater distances in the OF (F_(1,41)_ = 3.181, p = 0.082, Fig 4C), and displayed significantly more rearing behaviour in the NC in comparison to W3 mice (F_(1,41)_ = 5.888, p = 0.020, Fig 4D). The distance travelled on the EPM, however, was not affected by weaning age (F_(1,41)_ = 0.097, p = 0.757). And also concerning nest building performance, no effect of weaning age on nest quality after 7 h was detected (F_(1,41)_ = 1.995, p = 0.165, Table 3). Taken together, W4 mice thus behaved less anxious in the OF and more explorative in both the OF and the NC in comparison to their W3 conspecifics, while no overall differences occurred on the EPM and in the Nest Test (NT).

**Table 3:** Behavioural performance of W3 and W4 males and females.

	W3						W4						Statistics (p-values)		
	Males			Females			Males			Females			Weaning age	Sex	Weaning age*sex
	Mean	±	SEM	Mean	±	SEM	Mean	±	SEM	Mean	±	SEM			
EPM, distance travelled [m]	6.098	±	0.611	7.849	±	0.562	6.632	±	0.431	6.977	±	0.547	0.757	*0*.*060*	0.202
EPM, open arm entries (relative)	0.361	±	0.048	0.446	±	0.031	0.452	±	0.029	0.392	±	0.027	0.604	0.720	**0.049**
EPM, open arm time (relative)	0.328	±	0.065	0.483	±	0.052	0.417	±	0.049	0.386	±	0.049	0.943	0.278	0.102
EPM, open arm distance [m]	1.358	±	0.312	2.536	±	0.422	1.804	±	0.204	1.735	±	0.326	0.896	*0*.*072*	**0.036**
OF, distance [m]	26.697	±	1.408	32.864	±	1.789	31.473	±	1.755	34.060	±	1.655	*0*.*082*	**0.012**	0.291
OF, centre entries [#]	9.583	±	1.328	12.400	±	1.796	13.231	±	0.907	15.600	±	0.884	**0.010**	**0.046**	0.860
OF, centre time [s]	17.750	±	2.225	19.330	±	3.365	25.723	±	3.708	29.120	±	2.559	**0.005**	0.319	0.696
OF, distance travelled in centre [m]	2.683	±	0.353	3.496	±	0.462	3.609	±	0.248	4.728	±	0.346	**0.004**	**0.009**	0.665
NC, rearing [#]	53.167	±	3.047	60.300	±	3.997	60.923	±	3.207	71.600	±	5.528	**0.020**	**0.029**	0.654
NT, nest quality after 7h (score)	1.125	±	0.223	2.350	±	0.259	1.462	±	0.250	2.750	±	0.310	0.165	**0.000**	0.904

Summary of behavioural differences between male and female C57BL/6J mice weaned at either three weeks (W3) or four weeks (W4) of age. Behavioural parameters were assessed on the Elevated Plus Maze (EPM), in the Open Field (OF), in the Novel Cage (NC), and in the Nest Test (NT) at the age of about 20 weeks. Untransformed data are presented as means ± SEM and statistical findings of the 2-way ANOVAs are summarized by means of p-values. Significant main or interaction effects are indicated by bold numbers, while statistical trends are indicated by italic numbers. Sample sizes: N_males_W3_ = 12, N_females_W3_ = 10, N_males_W4_ = 13, N_females_W4_ = 10.

**Fig 5:**
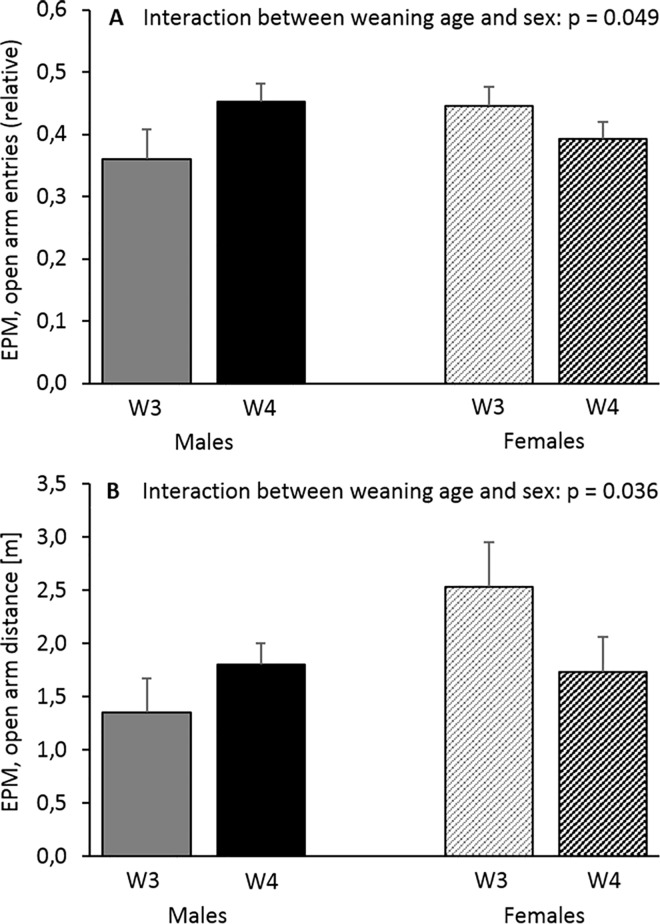
Performance of male and female C57BL/6J mice weaned after either three weeks (W3) or four weeks (W4) of age on the Elevated Plus Maze (EPM) at the age of about 20 weeks. (**A**) Relative number of open arm entries, and (**B**) distance travelled on the open arms. While W4 males displayed less anxiety-like behaviour than W3 males, it was the other way round in females. Data are presented as means + SEM. Statistics: 2-way ANOVA, sample sizes: N_males_W3_ = 12, N_females_W3_ = 10, N_males_W4_ = 13, N_females_W4_ = 10.

Interestingly, two interaction effects between sex and weaning age were detected that modulated anxiety-like behaviour on the EPM (Table 3). While in males the picture was the same as for anxiety-like behaviour in the Open Field (i.e. W4 mice entered the open arms of the EPM more often (F_(1,41)_ = 4.106, p = 0.049, Fig 5A) and travelled greater distances there than W3 mice (F_(1,41)_ = 4.702, p = 0.036, Fig 5B)), it was the other way round in females. In the other tests, however, no such interaction effects were found.

In addition to weaning age effects, several effects of sex were detected on anxiety-like behaviour, exploratory locomotion, and nest building performance (Table 3). Females behaved significantly less anxious and more explorative than males in the OF (centre entries: F_(1,41)_ = 4.239, p = 0.046; distance travelled in the centre: F_(1,41)_ = 7.584, p = 0.009; total distance: F_(1,41)_ = 6.833, p = 0.012), performed significantly more rearing behaviour in the NC (F_(1,41)_ = 5.143, p = 0.029), and built nests of higher quality in the NT (F_(1,41)_ = 23.235, p < 0.001). On the EPM, however, solely statistical trends were detected, indicating that females travelled overall greater distances both on the complete maze (F_(1,41)_ = 3.737, p = 0.060) and on the open arms (F_(1,41)_ = 3.415, p = 0.072). So again, females were characterized by more exploratory locomotion and less anxiety-like behaviour than their male conspecifics.

### Behavioural stability over time in W3 and W4 offspring

**Fig 6:**
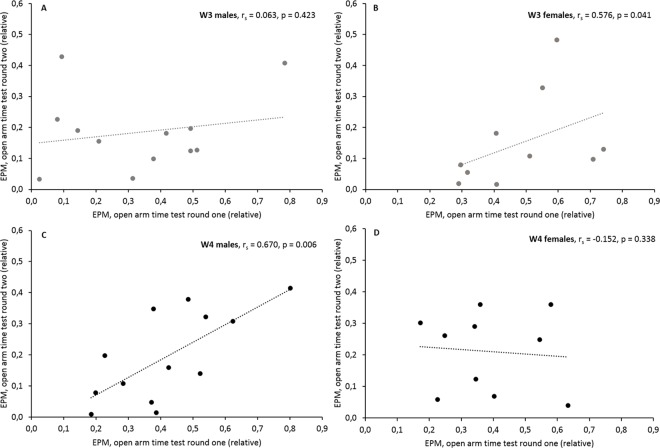
Behavioural stability over time in the time spent on the open arms of the Elevated Plus Maze (EPM) in male and female mice weaned after either three (W3) or four (W4) weeks. Correlations between test round I (20 weeks of age) and test round II (28 weeks of age) are separately presented for all four groups: (**A**) W3 males, (**B**) W3 females, (**C**) W4 males, and (**D**) W4 females. While there were significant correlations in W3 females and W4 males, no correlations were found in W3 males and W4 females. Statistics: Spearman’s rank correlation coefficient (1-tailed). Sample sizes: N_males_W3_ = 12, N_females_W3_ = 10, N_males_W4_ = 13, N_females_W4_ = 10.

Behavioural stability was investigated by repeating the series of behavioural tests at in interval of eight weeks during full adulthood. The analysis was then performed separately in male and female mice weaned after either three or four weeks of age. Interestingly, behavioural stability was highly dependent on the combination of weaning age and sex. While no significant correlations across time were detected in males of the W3 group (Spearman’s rank correlation coefficient, 1-tailed, r_s_ = -0.341–0.392, p = 0.139–0.441), statistical analyses revealed correlations in seven out of ten parameters in W3 females (Table 4; one example is illustrated in Fig 6). Significant correlations in the behaviour between test round I and II were thus found in this group with respect to anxiety-like behaviour in the OF (centre entries: r_s_ = 0.612, p = 0.030; centre time: r_s_ = 0.661, p = 0.019; distance travelled in the centre: r_s_ = 0.758; p = 0.006) and on the EPM (relative open arm time: r_s_ = 0.576, p = 0.041, Fig 6B; open arm distance: r_s_ = 0.661, p = 0.019) as well as with respect to nest building performance in the NT (r_s_ = 0.640, p = 0.023). Furthermore, a statistical trend was found for the relative open arm entries on the EPM (r_s_ = 0.486, p = 0.077), completing the picture of a high degree of behavioural stability in these mice. Measures used to describe exploratory locomotion on the EPM, in the OF, and in the NC, however, were not correlated across time (Table 4).

**Table 4:** Behavioural stability over time of W3 and W4 males and females.

	W3				W4			
	Males (n = 12)		Females (n = 10)		Males (n = 13)		Females (n = 10)	
	r_s_	p	r_s_	p	r_s_	p	r_s_	p
EPM, distance travelled [m]	-0.189	0.278	0.333	0.173	0.484	**0.047**	0.394	0.130
EPM, open arm entries (relative)	0.165	0.305	0.486	*0*.*077*	0.422	*0*.*076*	0.219	0.272
EPM, open arm time (relative)	0.063	0.423	0.576	**0.041**	0.670	**0.006**	-0.152	0.338
EPM, open arm distance [m]	0.056	0.431	0.661	**0.019**	0.670	**0.006**	0.127	0.363
OF, distance [m]	0.392	0.104	0.297	0.202	0.286	0.172	0.527	*0*.*059*
OF, centre entries [#]	-0.048	0.441	0.612	**0.030**	0.363	0.111	-0.272	0.224
OF, centre time [s]	-0.070	0.415	0.661	**0.019**	0.747	**0.002**	0.438	0.103
OF, distance travelled in centre [m]	0.343	0.138	0.758	**0.006**	0.429	*0*.*072*	-0.333	0.173
NC, rearing [#]	0.102	0.376	0.433	0.106	-0.121	0.347	0.219	0.272
NT, nest quality after 7h [score]	-0.341	0.139	0.640	**0.023**	0.368	0.108	0.390	0.133

Behavioural stability over time in male and female C57BL/6J mice weaned at either three weeks (W3) or four weeks (W4) of age. Behavioural parameters were assessed on the Elevated Plus Maze (EPM), in the Open Field (OF), in the Novel Cage (NC), and in the Nest Test (NT). To investigate behavioural stability, tests were conducted twice with the first test round starting at the age of about 20 weeks and the second round starting at the age of about 28 weeks. Correlations were calculated between performance in the first and the second test round using Spearman’s rank correlation coefficients r_s_ (1-tailed). Significant correlations are indicated by bold numbers, while statistical trends are indicated by italic numbers. Sample sizes: N_males_W3_ = 12, N_females_W3_ = 10, N_males_W4_ = 13, N_females_W4_ = 10.

In the W4 group, the picture was exactly opposite to the W3 group. Here, no significant correlations across time were detected in females, except one statistical trend for the distance travelled in the OF (r_s_ = 0.527, p = 0.059), while in males six out of ten parameters were found to be correlated between the test rounds (Table 4; one example is illustrated in Fig 6). Thus, the more anxious W4 males behaved in test round I on the EPM (relative open arm entries: r_s_ = 0.422, p = 0.076; relative open arm time: r_s_ = 0.670, p = 0.006, Fig 6C; open arm distance: r_s_ = 0.670, p = 0.006) and in the OF (centre time: r_s_ = 0.747, p = 0.002; centre distance: r_s_ = 0.429, p = 0.072), the more anxious they were in the second test round. Similarly, exploratory locomotion on the EPM, as measured by the distance travelled on the maze, was stable across time in this group (r_s_ = 0.484, p = 0.047). By contrast, no behavioural stability was detected in measures of exploratory locomotion in the OF and in the NC as well as in nest building performance in the NT (Table 4).

## Discussion

In the present study, we provide evidence for a long-lasting influence of weaning age on the behavioural phenotype of mice in full adulthood. In particular, mice weaned after three weeks of age (W3) behaved more anxious and less explorative in the Open Field (OF) and in the Novel Cage (NC) than mice weaned after four weeks of age (W4). In the Nest Test (NT), no difference in nest building performance was detected and on the Elevated Plus Maze (EPM), a weaning age dependent sexual dimorphism was observed. While ‘late weaning’ was associated with a reduction of anxiety-like behaviour on the EPM in males, it was the other way round in females. Interestingly, differences at the group level were accompanied by differences in the stability of behavioural expressions over time. Females weaned after three weeks of age were characterized by a high degree of behavioural stability over time, while this was not the case for W4 females. In males, however, the pattern was exactly the opposite. Here, males weaned after four weeks of age expressed a remarkable degree of stability over time, while behaviours in test rounds I and II were not at all correlated in W3 males.

### Maternal behaviour

Patterns of maternal care were very similar to those described previously by Curley and colleagues [26]. Nursing in C57BL/6J mice peaked around day 19 postpartum and then decreased gradually, while behaviours such as resting with body contact or self-grooming increased until the end of the observation period on postnatal day 28. Furthermore, while the frequency of drinking only slightly decreased over time, the frequency of eating dropped markedly between days 20 and 21. As pups start to eat solid food around this time, the dams’ behavioural changes may correspond to the nutritional demands of their pups (for review see [6]).

Nursing never disappeared completely in the present experiment, which is in contrast to early studies in house mice, showing that weaning is fully completed at the age of 23 days as judged from a drop in nursing activity to less than 1% [7]. However, the analysis of weaning weight data revealed that W3 pups reached an average weaning weight that was clearly below the reported natural weaning weight in house mice [6, 7]. The low but constant nursing frequencies throughout week 4 may therefore reflect an ongoing milk demand in mice that have not yet reached their optimal weaning weight [45]. The observed nursing frequencies may therefore result from the offspring shifting from passive nursing (initiated by the mother) to active sucking attempts [7].

Overall, maternal care data clearly show that the process of weaning took place gradually during the 4^th^ week postpartum and was characterized by marked changes in the dams’ behaviour. With respect to our research aim, W3 and W4 offspring thus not only experienced different housing and social environments, but also different amounts of maternal care and milk intake. Since variations in maternal care have long been discussed to be crucially involved in shaping the behavioural profile of the offspring [14, 17], differences in the maternal environment in the 4^th^ week postpartum may explain or at least contribute to some of the observed changes in later life. In this context, gene-by-environment interactions or epigenetic modifications to the genome may play a crucial role in mediating the behavioural phenotype as well as the consistency of behavioural expressions over time in W3 and W4 mice [14, 17].

### Weaning age and body weight development

Physical development of W3 and W4 offspring was monitored by regular body weight determinations over the course of the experiment. Interestingly, the initially lighter W3 animals caught up body weight rapidly between postnatal weeks 6 and 12 in such a way that their average adult weights were slightly above those of the W4 conspecifics. Since an artificially induced weaning after three weeks is associated with a sudden termination of the mother-infant relationship, it may represent an adverse experience during an early postnatal phase. If so, body weight changes are in line with previous findings in mice, showing that mildly adverse experiences during different stages of life lead to an increase in body weights afterwards. For example, exposure to olfactory cues of unfamiliar adult males during the prenatal and suckling period caused significantly higher body weights at the time of weaning compared to a control treatment [46]. Similarly, loser experiences as compared to mating experiences during adolescence and in later life were associated with higher body weights afterwards [38, 47].

### Weaning age and behaviour in adulthood

Several studies investigating the effects of ‘early weaning’ on the later behavioural profile of mice revealed increases in anxiety-like behaviour and locomotor activity in mice weaned after 14 or 15 days in comparison to mice weaned after 21 days (e.g., [19–21]). Similar results were obtained in rats, where the removal of the mother at postnatal day 15 or 16 was also found to elevate levels of anxiety-like behaviour and general activity [48, 49]. These findings indicate that premature weaning may indeed exert effects on the later behavioural profile similar to those seen in early separation studies (for review see [50]). So far, relatively little is known about effects of ‘late weaning’. Some first results in mice and rats indicate, however, that the removal from the mother at later time-points during the development may also induce changes in the behavioural profile of male and female offspring [26, 51, 52]. In line with these findings, differences in weaning age were associated with some differences in anxiety-like behaviour and exploratory locomotion in adulthood in the present study. W4 mice were clearly less anxious and more explorative than W3 mice in the OF and in the NC, but not on the EPM. Thus, although differences in anxiety-like behaviour could not be detected in all tests, the time point of weaning seems to be critically involved in shaping aspects of the behavioural profile in later life. In particular, an increasing delay of weaning from 14 to 28 days of age seems to be associated with a stepwise reduction of anxiety-like behaviour displayed in novel test situations [19, 20]. However, since OF and EPM did not result in consistent findings, this reduction may concern only specific aspects of mouse anxiety-like behaviour. Overall, these findings fit well into the picture of a modulating effect of weaning, although they are in contrast to Curley and colleagues [26], who did not observe any differences in anxiety-like behaviour between mice weaned after either 21 or 28 days of age. Notably, Curley and colleagues tested the offspring between postnatal days 50 and 74, i.e. during late adolescence/early adulthood, while experiments in the present study were conducted between PNDs 139–146, i.e. during full adulthood. A previous study in mice has shown that effects of early-life differences may become more visible in full adulthood than in adolescence [37]. Thus, when behavioural testing is conducted too early in life, differences in anxiety-like behaviour may not be detected.

### Sex differences in behaviour in adulthood

Concerning the effects of sex, several behavioural differences between male and female C57BL/6J mice were detected during full adulthood. Overall, females behaved less anxious and more explorative than males on the EPM, in the OF, and in the NC, and were, furthermore, found to build nests of higher quality in the NT. Robust sex differences have frequently been described for numerous mouse strains, including the here used C57BL/6J strain (e.g., [53–56]). Although there is a general disagreement about the direction of these differences, our results confirm the occurrence of such sex-specific differences in laboratory mouse behaviour.

Furthermore, we detected significant interactions between weaning age and sex modulating anxiety-like behaviour on the EPM. While in males, ‘late weaning’ was associated with a reduction of anxiety-like behaviour, it was the other way round in females. Interestingly, such sex-specific effects have also been described before in the context of weaning studies (e.g., [26, 51]). For example, Curley and colleagues found elevated levels of exploratory locomotion in the OF in W4 males, but reduced exploratory behaviour in W4 females [26]. Similarly, Laviola and Dell’Omo did not detect any differences in activity between ‘early weaned’ and ‘regularly weaned’ female rats, while ‘early weaned’ males were more active than those weaned regularly [21]. As it has been suggested before, weaning age may thus be a useful variable for systematically addressing the development of a behavioural sexual dimorphism in mice [26].

### Weaning age and behavioural stability over time in adulthood

Most interestingly, a sex-specific pattern was not only found in the behaviour at the group level, but also with respect to individual behavioural stability over time. While W3 females and W4 males displayed a remarkable degree of behavioural stability over time, no such patterns were found in W3 males and W4 females. Consistent inter-individual differences that are stable over time and/or across contexts have mainly been discussed with respect to ‘animal personalities’ that have been described for a wide range of taxa (e.g., [27]). Recently, such ‘personalities’ have also been detected in genetically identical mice that live in identical housing environments [57, 58]. However, despite the increasing interest in the study of stable inter-individual differences, relatively little is known about ontogenetic factors that may influence the stability of such traits.

There is evidence from numerous studies that males and females, both in humans and non-human animals, can differ in the mean level of their behavioural expressions, while hardly any studies have addressed sex differences in the consistency of behaviour in a systematic way (for review see [59]). So far, Dingemanse and colleagues investigated sex differences in the repeatability of exploratory behaviour in great tits, but did not find any differences between males and females [60]. By contrast, Holder and colleagues found that male Midas cichlids are more consistent in their tendency to behave aggressively over time than females [61]. Adding to these first findings, the consistency in anxiety-like behaviour, exploratory locomotion, and nest building performance in adulthood differed between male and female mice in the present study, but was additionally modulated by an ecologically relevant factor, i. e. weaning age.

In rodent ecology, dispersal behaviour has long been recognized as one of the key life history experiences, as it is usually associated with a radical shift in the environmental conditions an individual lives in [62]. Successful dispersal may therefore require a high degree of behavioural flexibility rather than behavioural stability, favouring those individuals that are able to adjust the behaviour according to the changing conditions. Among house mice, dispersal is known to be sex-dependent with male mice dispersing earlier and more frequently than female mice [63, 64]. Furthermore, several life history factors as well as ecological constraints have been discussed to delay or prevent dispersal completely [65]. The observed differences in behavioural stability over time may therefore reflect differences in dispersal strategies between male and female mice that were additionally shaped by different weaning experiences. In principle, one may therefore assume a framework as follows: Ecological conditions, such as low/high food availability or low/high population densities, favour different weaning strategies that in turn are related to dispersal or philopatric behaviour in a sex-specific manner, and thus result in different degrees of behavioural stability in adulthood. However, since the literature is scarce about these relationships and we did not take any direct measures of dispersal behaviour in the present study, we prefer to not speculate about these findings further.

## Conclusions

Weaning has frequently been emphasized as an important life history variable that modulates the behavioural profile in later life. We here confirm that profound behavioural effects of weaning age do exist, and furthermore provide first evidence for the impact of weaning age and sex on the consistency of behavioural expressions over time. While at the group level, ‘late weaning’ after four weeks was associated with reduced levels of anxiety-like behaviour and increased exploratory locomotion, the picture was far more complex for weaning age-related differences in behavioural stability over time. Here, different dispersal strategies in W3 and W4 males and females may explain the development of different degrees of behavioural stability among individuals.

## Supporting Information

S1 Table Average maternal care observed in the third week postpartum. Data are separately presented for each dam of the W3 and W4 groups.
(PDF)

S2 Table Offspring body weight measurements. Data are separately presented for each male or female individual of the W3 and W4 groups.
(PDF)

S3 Table Raw data resulting from behavioural testing in test round I. Data are separately presented for each male or female individual of the W3 and W4 groups (NC = Novel Cage, NT = Nest Test, EPM = Elevated Plus Maze, OF = Open Field).
(PDF)

S4 Table Raw data resulting from behavioural testing in test round II. Data are separately presented for each male or female individual of the W3 and W4 groups (NC = Novel Cage, NT = Nest Test, EPM = Elevated Plus Maze, OF = Open Field).
(PDF)
