# Supplementary material for: A Time to Wean? Impact of Weaning Age on Anxiety-Like Behaviour and Stability of Behavioural Traits in Full Adulthood
Source: PLoS One. 2016 Dec 8;11(12):e0167652. doi: 10.1371/journal.pone.0167652 (PMC5145172; doi:10.1371/journal.pone.0167652)
Supplement: S1 Table — Data are separately presented for each dam of the W3 and W4 groups. (PDF) [file pone.0167652.s001.pdf]

S1 Table.

Dam ID	Weaning age	Batch	Litter size	Licking/ grooming	Contact	Nest building	Self grooming	Eating	Drinking	Nursing
1127	W3	1	7	0.007	0.082	0.000	0.089	0.286	0.055	0.311
1128	W3	1	9	0.023	0.038	0.002	0.100	0.200	0.071	0.543
1129	W3	1	8	0.021	0.054	0.004	0.048	0.288	0.041	0.450
1131	W3	1	4	0.041	0.052	0.000	0.122	0.224	0.039	0.326
1134	W3	1	5	0.036	0.070	0.005	0.100	0.261	0.061	0.489
1137	W3	1	4	0.027	0.054	0.002	0.152	0.143	0.047	0.348
1265	W3	2	5	0.014	0.034	0.004	0.073	0.304	0.034	0.359
1266	W3	2	7	0.020	0.048	0.000	0.084	0.343	0.070	0.427
1269	W3	2	5	0.016	0.084	0.002	0.109	0.255	0.029	0.370
1270	W3	2	8	0.025	0.048	0.000	0.046	0.321	0.120	0.471
1272	W3	2	7	0.018	0.086	0.000	0.095	0.329	0.070	0.332
1125	W4	1	7	0.020	0.123	0.002	0.079	0.274	0.047	0.390
1126	W4	1	6	0.030	0.107	0.007	0.121	0.173	0.030	0.432
1130	W4	1	8	0.027	0.061	0.002	0.104	0.207	0.061	0.421
1132	W4	1	6	0.025	0.065	0.002	0.161	0.243	0.038	0.242
1136	W4	1	6	0.021	0.086	0.020	0.075	0.364	0.050	0.400
1138	W4	1	9	0.020	0.063	0.002	0.063	0.327	0.079	0.366
1267	W4	2	9	0.013	0.041	0.002	0.070	0.332	0.080	0.479
1268	W4	2	8	0.013	0.070	0.002	0.102	0.339	0.063	0.241
1273	W4	2	7	0.007	0.100	0.000	0.064	0.379	0.080	0.373
1274	W4	2	5	0.021	0.050	0.002	0.107	0.314	0.025	0.266
