# Supplementary material for: A Time to Wean? Impact of Weaning Age on Anxiety-Like Behaviour and Stability of Behavioural Traits in Full Adulthood
Source: PLoS One. 2016 Dec 8;11(12):e0167652. doi: 10.1371/journal.pone.0167652 (PMC5145172; doi:10.1371/journal.pone.0167652)
Supplement: S2 Table — Data are separately presented for each male or female individual of the W3 and W4 groups. (PDF) [file pone.0167652.s002.pdf]

S2 Table.

Animal ID	Sex	Weaning age	Batch	Weaning weight	Weight in week 6	Weight in week 12	Weight in week 18	Weight in week 24	Weight in week 30
1163	male	W3	1	7.20	18.00	26.00	30.00	31.10	32.20
1168	female	W3	1	7.80	15.10	19.80	20.70	23.30	24.00
1169	male	W3	1	6.90	19.00	27.20	30.00	30.90	32.20
1182	male	W3	1	10.00	20.00	28.90	31.20	31.30	32.70
1185	female	W3	1	7.10	13.60	19.90	22.10	23.30	24.90
1188	female	W3	1	7.70	14.70	18.20	20.40	21.40	22.60
1190	male	W3	1	7.60	15.30	22.00	26.30	28.60	28.90
1195	female	W3	1	7.60	14.40	19.30	21.30	21.90	23.10
1198	male	W3	1	8.10	16.10	26.30	28.10	30.60	31.10
1201	female	W3	1	7.60	15.70	20.90	22.70	22.90	22.80
1202	male	W3	1	8.60	16.20	26.00	29.50	31.60	32.50
1206	female	W3	1	8.20	15.50	20.00	21.20	22.90	22.80
1209	male	W4	1	16.40	20.00	27.20	28.90	31.20	33.20
1214	female	W4	1	12.00	15.20	18.50	20.00	21.60	22.40
1217	female	W4	1	10.70	14.30	17.70	19.10	19.40	21.40
1219	male	W4	1	15.00	18.10	23.10	24.40	27.50	28.60
1223	female	W4	1	13.60	15.50	20.20	21.90	23.70	23.10
1226	male	W4	1	15.80	19.00	26.20	28.10	30.80	32.50
1229	male	W4	1	14.50	17.50	25.50	28.20	29.40	31.10
1230	female	W4	1	14.60	17.40	20.50	23.00	25.10	26.20
1234	male	W4	1	14.80	19.60	26.00	27.60	28.40	29.40
1236	female	W4	1	12.90	15.80	19.30	21.20	21.50	22.90
1239	female	W4	1	12.60	15.90	19.80	21.30	21.50	23.50
1242	male	W4	1	14.30	19.00	26.60	29.30	29.80	30.80
1245	male	W4	1	14.40	19.00	26.10	29.40	30.30	32.10
1248	female	W4	1	14.10	16.70	20.20	21.00	22.00	22.40
1287	female	W3	2	8.50	16.00	21.90	23.30	24.50	24.80
1290	male	W3	2	8.30	17.50	27.70	31.50	32.20	33.00
1293	female	W3	2	7.10	12.90	18.10	20.10	20.60	21.70
1297	male	W3	2	7.70	16.40	25.70	29.20	29.60	29.00

1300	male	W3	2	9.10	17.50	26.40	30.00	31.10	31.40
1303	male	W3	2	7.30	15.50	26.30	30.40	30.50	30.40
1306	female	W3	2	6.70	14.30	19.90	22.80	22.50	23.60
1309	male	W3	2	8.70	17.70	26.60	28.30	30.00	31.90
1312	male	W3	2	4.10	10.00	21.60	26.60	27.90	28.90
1313	female	W3	2	8.90	15.80	20.60	23.00	23.10	25.10
1318	male	W4	2	12.20	15.60	25.60	28.10	29.10	30.20
1321	male	W4	2	13.20	14.10	24.80	29.50	29.90	30.90
1324	female	W4	2	10.80	14.50	19.30	22.70	22.50	22.80
1327	male	W4	2	9.90	13.00	22.70	28.50	28.70	29.70
1330	male	W4	2	14.50	18.70	25.80	29.40	30.30	32.50
1335	male	W4	2	12.00	14.50	27.20	29.70	30.60	30.60
1336	female	W4	2	12.90	15.30	20.10	21.50	22.70	22.70
1339	female	W4	2	12.00	14.80	18.50	21.60	20.70	21.90
1342	male	W4	2	14.00	18.40	26.30	28.90	30.40	31.40
