# Supplementary material for: A Time to Wean? Impact of Weaning Age on Anxiety-Like Behaviour and Stability of Behavioural Traits in Full Adulthood
Source: PLoS One. 2016 Dec 8;11(12):e0167652. doi: 10.1371/journal.pone.0167652 (PMC5145172; doi:10.1371/journal.pone.0167652)
Supplement: S3 Table — Data are separately presented for each male or female individual of the W3 and W4 groups (NC = Novel Cage, NT = Nest Test, EPM = Elevated Plus Maze, OF = Open Field). (PDF) [file pone.0167652.s003.pdf]

S3 Table.

Animal ID	Sex	Weaning age	Batch	NC1 Rearing	NT1 Score	EPM1 Distance	EPM1 Open arm entries	EPM1 Open arm time	EPM1 Open arm distance	OF1 Distance	OF1 Center entries	OF1 Center time	OF1 Center distance
1163	male	W3	1	77	0	2.39	0.63	0.78	1.00	22.02	7	12.2	1.96
1168	female	W3	1	63	2.5	6.35	0.41	0.41	1.97	36.27	8	14.5	2.58
1169	male	W3	1	51	0.5	5.43	0.21	0.21	1.01	27.57	9	16.9	2.12
1182	male	W3	1	42	0.5	3.60	0.10	0.02	0.03	21.96	5	7.5	0.87
1185	female	W3	1	45	2.5	7.93	0.46	0.41	2.43	30.45	7	9.5	2.10
1188	female	W3	1	64	3	9.95	0.57	0.74	5.19	30.02	14	14.4	2.96
1190	male	W3	1	39	2	5.12	0.13	0.09	0.54	34.86	8	19.1	3.15
1195	female	W3	1	69	3	8.85	0.52	0.60	3.78	29.37	14	42.2	4.80
1198	male	W3	1	54	1	4.41	0.36	0.31	0.63	22.46	7	25.1	2.29
1201	female	W3	1	56	3	8.02	0.35	0.32	1.59	35.85	15	14.6	3.75
1202	male	W3	1	47	2.5	7.81	0.48	0.42	2.05	33.24	14	22.5	4.00
1206	female	W3	1	81	2	6.04	0.61	0.51	1.85	30.24	13	21.7	4.27
1209	male	W4	1	65	2	4.01	0.67	0.80	2.28	21.98	7	12.6	1.93
1214	female	W4	1	64	2	3.56	0.25	0.23	0.61	41.81	18	23.3	5.51
1217	female	W4	1	90	3.5	6.86	0.48	0.63	2.58	40.66	21	27.2	6.00
1219	male	W4	1	62	0.5	6.06	0.61	0.62	2.25	23.97	13	42.2	3.51
1223	female	W4	1	59	2.5	6.72	0.42	0.40	1.28	29.16	15	27.1	4.38
1226	male	W4	1	83	3	7.62	0.32	0.23	1.52	37.01	12	10.7	2.87
1229	male	W4	1	66	2.5	6.60	0.41	0.54	2.19	40.39	21	26.4	5.76
1230	female	W4	1	69	4	6.24	0.50	0.25	0.71	32.03	15	34.9	3.25
1234	male	W4	1	67	2.5	6.38	0.41	0.20	1.10	28.87	14	55.5	4.31
1236	female	W4	1	76	3	6.54	0.35	0.54	1.94	28.13	11	34.9	3.98
1239	female	W4	1	90	4	10.03	0.50	0.58	4.00	38.78	15	27.4	4.35
1242	male	W4	1	74	0.5	4.72	0.44	0.52	1.55	33.66	16	26.0	3.95
1245	male	W4	1	70	1.5	5.01	0.28	0.18	0.21	29.38	15	24.6	3.65
1248	female	W4	1	74	1.5	7.06	0.38	0.36	1.10	33.29	17	44.4	6.06
1287	female	W3	2	61	1	9.48	0.33	0.30	1.76	38.17	16	16.2	3.70
1290	male	W3	2	58	0.5	6.34	0.37	0.51	1.47	24.90	11	17.5	2.73
1293	female	W3	2	73	1.5	9.36	0.48	0.55	4.07	44.11	24	33.9	6.48
1297	male	W3	2	63	0.5	10.12	0.44	0.49	3.64	30.93	20	33.0	5.16

1300	male	W3	2	58	1	5.74	0.35	0.08	0.13	25.75	9	16.1	2.46
1303	male	W3	2	44	1.5	7.93	0.60	0.49	2.81	20.86	2	4.2	0.88
1306	female	W3	2	51	1.5	8.19	0.36	0.29	1.24	29.66	9	16.7	2.98
1309	male	W3	2	47	2	6.77	0.42	0.38	1.88	24.03	12	22.1	3.42
1312	male	W3	2	58	1.5	7.52	0.24	0.14	1.13	31.78	11	16.8	3.16
1313	female	W3	2	40	3.5	4.33	0.38	0.71	1.48	24.51	4	9.6	1.35
1318	male	W4	2	56	0.5	9.28	0.47	0.49	3.29	30.23	14	21.6	4.17
1321	male	W4	2	51	1.5	7.80	0.48	0.39	2.05	28.54	12	24.5	3.43
1324	female	W4	2	41	1.5	9.03	0.36	0.34	2.13	31.36	13	25.9	5.41
1327	male	W4	2	52	0.5	8.80	0.48	0.38	2.24	39.98	12	14.4	3.38
1330	male	W4	2	56	0.5	6.63	0.40	0.37	1.85	40.32	14	15.9	3.77
1335	male	W4	2	50	2	7.49	0.48	0.42	1.57	24.77	10	42.4	2.91
1336	female	W4	2	97	2	6.18	0.28	0.17	1.02	37.46	14	14.1	3.01
1339	female	W4	2	56	3.5	7.54	0.40	0.35	1.99	27.93	17	32.0	5.32
1342	male	W4	2	40	1.5	5.83	0.44	0.28	1.36	30.05	12	17.6	3.27

S4 Table. Behavioural data, test round 2.

Animal	Sex	Weaning age	Batch	NC2 Rearing	NT2 Score	EPM2 Distance	EPM2 Open arm entries	EPM2 Open arm time	EPM2 Open arm distance	OF2 Distance	OF2 Center entries	OF2 Center time	OF2 Center distance
1163	male	EW	1	52	2	6.757	0.522	0.407	2.173	23.061	7	11.8	1.857
1168	female	EW	1	57	3	7.531	0.235	0.180	0.892	34.989	5	7.2	1.601
1169	male	EW	1	37	1	5.987	0.200	0.156	0.558	18.753	3	5.7	1.082
1182	male	EW	1	42	5	4.055	0.286	0.033	0.000	19.160	3	15.3	0.916
1185	female	EW	1	48	2	5.058	0.067	0.016	0.050	20.206	3	4.9	0.971
1188	female	EW	1	57	2.5	4.835	0.400	0.129	1.017	24.427	10	18.1	2.138
1190	male	EW	1	49	3	10.321	0.400	0.428	2.559	37.027	12	11.7	3.029
1195	female	EW	1	51	3	6.867	0.478	0.483	2.262	28.809	12	18.6	2.711
1198	male	EW	1	55	1.5	3.869	0.250	0.036	0.122	29.638	9	12.9	2.157
1201	female	EW	1	50	2.5	5.860	0.211	0.055	0.484	29.710	3	6.3	1.187
1202	male	EW	1	27	0.5	6.943	0.190	0.180	1.076	27.939	17	29.9	4.365
1206	female	EW	1	67	0.5	5.599	0.200	0.108	0.432	25.736	7	17.2	2.618
1209	male	LW	1	36	3	2.791	0.500	0.415	0.918	15.922	1	0.5	0.134
1214	female	LW	1	26	2	3.464	0.111	0.059	0.419	19.250	3	5.0	0.660
1217	female	LW	1	66	2.5	4.932	0.125	0.040	0.059	39.271	8	6.5	1.592
1219	male	LW	1	71	1.5	6.765	0.300	0.308	1.626	32.717	17	30.7	5.425
1223	female	LW	1	53	1	4.603	0.143	0.068	0.291	22.819	6	8.8	1.671
1226	male	LW	1	50	1.5	6.361	0.333	0.198	1.009	18.733	6	5.4	1.070
1229	male	LW	1	78	2.5	4.936	0.286	0.322	1.085	31.764	15	29.0	5.209
1230	female	LW	1	56	3.5	8.420	0.320	0.261	1.449	23.684	10	17.6	2.546
1234	male	LW	1	40	2	5.070	0.118	0.078	0.378	25.240	8	39.6	2.434
1236	female	LW	1	54	3	6.357	0.389	0.248	0.806	19.341	9	23.8	3.125
1239	female	LW	1	73	3.5	9.004	0.333	0.359	2.132	39.558	16	28.5	5.703
1242	male	LW	1	57	0.5	6.201	0.118	0.140	0.726	21.083	7	10.7	1.829
1245	male	LW	1	26	1	5.572	0.095	0.009	0.000	30.638	11	17.3	3.057
1248	female	LW	1	60	1.5	5.808	0.318	0.360	1.415	23.573	10	57.7	3.347
1287	female	EW	2	50	0.5	7.102	0.118	0.079	0.697	25.952	8	11.4	2.115
1290	male	EW	2	49	3	5.568	0.158	0.128	0.521	23.960	6	8.3	1.445
1293	female	EW	2	43	1	8.466	0.360	0.328	1.817	35.033	14	24.0	4.200
1297	male	EW	2	54	2	5.559	0.313	0.197	0.807	22.817	6	16.5	1.998

1300	male	EW	2	33	0.5	8.112	0.273	0.226	0.681	36.840	19	31.5	6.099
1303	male	EW	2	51	3	3.767	0.333	0.124	0.508	26.864	14	14.1	3.740
1306	female	EW	2	64	2	5.959	0.067	0.018	0.120	31.118	6	8.4	1.792
1309	male	EW	2	72	1	5.618	0.200	0.099	0.077	24.981	7	7.6	1.962
1312	male	EW	2	35	1.5	4.435	0.167	0.190	0.877	30.001	8	13.3	2.530
1313	female	EW	2	38	2	4.729	0.227	0.097	0.267	27.592	5	10.0	1.215
1318	male	LW	2	58	2	7.989	0.423	0.378	2.341	29.246	8	6.2	1.276
1321	male	LW	2	65	1	3.944	0.083	0.015	0.025	20.711	10	14.6	3.652
1324	female	LW	2	62	3	7.885	0.263	0.290	1.536	21.239	5	6.3	0.820
1327	male	LW	2	43	2	7.330	0.476	0.348	1.849	34.843	9	11.3	2.556
1330	male	LW	2	59	1.5	5.730	0.176	0.047	0.180	28.796	9	21.7	3.199
1335	male	LW	2	47	2.5	5.994	0.200	0.159	0.923	27.426	10	17.2	2.793
1336	female	LW	2	58	1	6.011	0.278	0.301	1.430	25.364	10	30.5	3.142
1339	female	LW	2	61	0.5	5.454	0.167	0.123	0.572	15.335	3	7.9	0.648
1342	male	LW	2	47	0.5	4.530	0.400	0.108	0.626	15.914	2	6.6	0.926
