# Supplementary material for: A Time to Wean? Impact of Weaning Age on Anxiety-Like Behaviour and Stability of Behavioural Traits in Full Adulthood
Source: PLoS One. 2016 Dec 8;11(12):e0167652. doi: 10.1371/journal.pone.0167652 (PMC5145172; doi:10.1371/journal.pone.0167652)
Supplement: S4 Table — Data are separately presented for each male or female individual of the W3 and W4 groups (NC = Novel Cage, NT = Nest Test, EPM = Elevated Plus Maze, OF = Open Field). (PDF) [file pone.0167652.s004.pdf]

S4 Table.

Animal ID	Sex	Weaning age	Batch	NC2 Rearing	NT2 Score	EPM2 Distance	EPM2 Open arm entries	EPM2 Open arm time	EPM2 Open arm distance	OF2 Distance	OF2 Center entries	OF2 Center time	OF2 Center distance
1163	male	W3	1	52	2	6.757	0.522	0.407	2.173	23.061	7	11.8	1.857
1168	female	W3	1	57	3	7.531	0.235	0.180	0.892	34.989	5	7.2	1.601
1169	male	W3	1	37	1	5.987	0.200	0.156	0.558	18.753	3	5.7	1.082
1182	male	W3	1	42	5	4.055	0.286	0.033	0.000	19.160	3	15.3	0.916
1185	female	W3	1	48	2	5.058	0.067	0.016	0.050	20.206	3	4.9	0.971
1188	female	W3	1	57	2.5	4.835	0.400	0.129	1.017	24.427	10	18.1	2.138
1190	male	W3	1	49	3	10.321	0.400	0.428	2.559	37.027	12	11.7	3.029
1195	female	W3	1	51	3	6.867	0.478	0.483	2.262	28.809	12	18.6	2.711
1198	male	W3	1	55	1.5	3.869	0.250	0.036	0.122	29.638	9	12.9	2.157
1201	female	W3	1	50	2.5	5.860	0.211	0.055	0.484	29.710	3	6.3	1.187
1202	male	W3	1	27	0.5	6.943	0.190	0.180	1.076	27.939	17	29.9	4.365
1206	female	W3	1	67	0.5	5.599	0.200	0.108	0.432	25.736	7	17.2	2.618
1209	male	W4	1	36	3	2.791	0.500	0.415	0.918	15.922	1	0.5	0.134
1214	female	W4	1	26	2	3.464	0.111	0.059	0.419	19.250	3	5.0	0.660
1217	female	W4	1	66	2.5	4.932	0.125	0.040	0.059	39.271	8	6.5	1.592
1219	male	W4	1	71	1.5	6.765	0.300	0.308	1.626	32.717	17	30.7	5.425
1223	female	W4	1	53	1	4.603	0.143	0.068	0.291	22.819	6	8.8	1.671
1226	male	W4	1	50	1.5	6.361	0.333	0.198	1.009	18.733	6	5.4	1.070
1229	male	W4	1	78	2.5	4.936	0.286	0.322	1.085	31.764	15	29.0	5.209
1230	female	W4	1	56	3.5	8.420	0.320	0.261	1.449	23.684	10	17.6	2.546
1234	male	W4	1	40	2	5.070	0.118	0.078	0.378	25.240	8	39.6	2.434
1236	female	W4	1	54	3	6.357	0.389	0.248	0.806	19.341	9	23.8	3.125
1239	female	W4	1	73	3.5	9.004	0.333	0.359	2.132	39.558	16	28.5	5.703
1242	male	W4	1	57	0.5	6.201	0.118	0.140	0.726	21.083	7	10.7	1.829
1245	male	W4	1	26	1	5.572	0.095	0.009	0.000	30.638	11	17.3	3.057
1248	female	W4	1	60	1.5	5.808	0.318	0.360	1.415	23.573	10	57.7	3.347
1287	female	W3	2	50	0.5	7.102	0.118	0.079	0.697	25.952	8	11.4	2.115
1290	male	W3	2	49	3	5.568	0.158	0.128	0.521	23.960	6	8.3	1.445
1293	female	W3	2	43	1	8.466	0.360	0.328	1.817	35.033	14	24.0	4.200
1297	male	W3	2	54	2	5.559	0.313	0.197	0.807	22.817	6	16.5	1.998

1300	male	W3	2	33	0.5	8.112	0.273	0.226	0.681	36.840	19	31.5	6.099
1303	male	W3	2	51	3	3.767	0.333	0.124	0.508	26.864	14	14.1	3.740
1306	female	W3	2	64	2	5.959	0.067	0.018	0.120	31.118	6	8.4	1.792
1309	male	W3	2	72	1	5.618	0.200	0.099	0.077	24.981	7	7.6	1.962
1312	male	W3	2	35	1.5	4.435	0.167	0.190	0.877	30.001	8	13.3	2.530
1313	female	W3	2	38	2	4.729	0.227	0.097	0.267	27.592	5	10.0	1.215
1318	male	W4	2	58	2	7.989	0.423	0.378	2.341	29.246	8	6.2	1.276
1321	male	W4	2	65	1	3.944	0.083	0.015	0.025	20.711	10	14.6	3.652
1324	female	W4	2	62	3	7.885	0.263	0.290	1.536	21.239	5	6.3	0.820
1327	male	W4	2	43	2	7.330	0.476	0.348	1.849	34.843	9	11.3	2.556
1330	male	W4	2	59	1.5	5.730	0.176	0.047	0.180	28.796	9	21.7	3.199
1335	male	W4	2	47	2.5	5.994	0.200	0.159	0.923	27.426	10	17.2	2.793
1336	female	W4	2	58	1	6.011	0.278	0.301	1.430	25.364	10	30.5	3.142
1339	female	W4	2	61	0.5	5.454	0.167	0.123	0.572	15.335	3	7.9	0.648
1342	male	W4	2	47	0.5	4.530	0.400	0.108	0.626	15.914	2	6.6	0.926
